# Transcriptome-wide analysis reveals sequence selection to avoid mRNA aggregation in *E. coli*

**DOI:** 10.1101/2025.09.19.677370

**Published:** 2025-09-20

**Authors:** Marco Todisco, Ankur Jain

**Affiliations:** 1 Whitehead Institute for Biomedical Research, Cambridge, MA, 02142, USA; 2 Department of Biology, Massachusetts Institute of Technology, Cambridge, MA, 02139, USA.

**Keywords:** RNA-RNA interactions, Biomolecular condensates, RNA Aggregation, Transcriptome Dynamics, Molecular Evolution, Biological Sciences (Biophysics and Computational Biology), Physical Sciences (Biophysics and Computational Biology)

## Abstract

The stability of RNA base pairing and its limited four-letter code create an intrinsic potential for promiscuous RNA-RNA interactions. *In vitro*, such interactions drive RNA to self-assemble into aggregates. This raises a fundamental unanswered question: within a confined cellular volume at physiological mRNA abundances, how much aggregation would arise from sequence-encoded chemistry alone? Here, we establish this baseline with large-scale kinetic simulations of the *E. coli* transcriptome. Our simulations reveal that sequence-encoded base-pairing energetics is sufficient to generate a dynamic network of large aggregates, organized by long, multivalent mRNA hubs. Strikingly, evolutionary analysis shows that native *E. coli* sequences exhibit clear signatures of selection to counteract this propensity: they fold more stably, minimize unstructured regions, and form weaker intermolecular contacts than dinucleotide-preserving controls. These findings demonstrate that maintaining transcriptome solubility has been a significant, previously unrecognized constraint shaping genome evolution, and provide a new lens to interpret cellular RNA management.

## Introduction

Messenger RNAs couple genetic information to protein output. This decoding hinges on base-pairing between mRNA codons and tRNA anticodons, that unambiguously map nucleotide triplets to amino acids. The same base-pairing interactions that allow DNA-to-protein information transfer, also form the cornerstone of cellular regulation: in bacteria, rRNA-mRNA complementarity (Shine-Dalgarno sequence) helps position ribosomes on start sites and small regulatory RNAs modulate mRNA stability and translation. Likewise, in eukaryotes, microRNAs repress mRNAs and snRNAs/snoRNAs guide RNA processing and site-specific modification.

Beyond these well-defined regulatory roles, recent high throughput studies have also shown extensive mRNA-mRNA association *in vivo* (1, 2). The biological function of these interactions – if any – remains largely unexplored. Studies on condensates such as stress granules (3, 4), TIS granules (5) and RNA aggregates associated with repeat-expansion disorders (6–8) implicate mRNA-mRNA interactions in both formation and maintenance of these bodies, although experimentally decoupling RNA’s intrinsic role from protein scaffolding is challenging. Notably, total mRNA from yeast, purified and freed from proteins, readily self-assembles *in vitro* into droplets or tangles that resemble stress granules in composition (3). Taken together, these observations suggest that mRNA-mRNA interactions *in vivo* are both plausible and potentially consequential.

These observations motivate a fundamental question: given only sequence, abundance, and cellular volume, how much intermolecular mRNA pairing should one expect? RNA base pairing has been systematically studied since the 1970s (9, 10), establishing a robust understanding of RNA folding and pairwise interactions *in vitro*. However, scaling these principles to predict the system-level dynamics when many RNAs interact simultaneously remains challenging (11–18).

Here we quantify the sequence-encoded, RNA-only aggregation pressure of the *E. coli* transcriptome, establishing an upper bound on intermolecular mRNA pairing in the absence of translation and RNA-binding proteins (RBPs). This baseline allows us to (i) measure how much aggregation RNA chemistry alone would produce at physiological concentrations, and (ii) ask whether native mRNA sequences are evolutionarily tuned to reduce that pressure. Using thermodynamic models of RNA base pairing, we first identify unstructured regions available for intermolecular association in each transcript. These sites, combined with known abundances and cellular volumes, are used to parameterize stochastic kinetic simulations that capture the dynamic association and dissociation of mRNAs. We focus exclusively on mRNAs because rRNAs and tRNAs are expected to be stably folded, extensively bound by proteins, and lack widespread complementarity to mRNAs (19).

Our analysis revealed the existence of a dynamic steady state where thousands of mRNA interactions break and form on a fast timescale. Since individual mRNA molecules can interact with multiple partners simultaneously, a large fraction of the *E. coli* transcriptome is expected to coalesce into aggregates containing hundreds of mRNAs. These clusters are organized by long, high-valency mRNAs, mirroring the enrichment of long RNAs in eukaryotic stress granules. To test whether evolution has shaped sequences to minimize this aggregation, we compared native *E. coli* mRNAs to dinucleotide-shuffled controls. We found that the naturally occurring sequences exhibit more stable intramolecular folding and form significantly weaker intermolecular interactions when compared to their randomized counterparts. Our work highlights how the fundamental chemistry of RNA imposes physical constraints that have shaped the evolution of genetic information and necessitate mechanisms to maintain transcriptome solubility.

## Results

### 1. Identifying the intermolecular interaction potential of the E. coli transcriptome

We developed a computational model to test whether the cellular mRNA pool is expected to form extensive intermolecular base pairs, focusing on the *E. coli* transcriptome for tractability and relevance. *E. coli* grown in Luria-Bertani (LB) medium contain an estimated 7,800 mRNA/cell (20) in an intracellular volume of 6.7 × 10^−10^ μL/cell (21), corresponding to a concentration of roughly 20 μM of RNA. Transcript abundances were taken from previous RNA-seq datasets (22), allowing us to model physiologically relevant RNA concentrations. RNA molecules intrinsically fold into patterns of paired and unpaired segments such as hairpin loops, bulges, and internal loops. Previous studies have shown that network-forming RNAs are enriched in unstructured stretches (USs): extended single-stranded tracts that are available for hybridization and hypothesized to drive mRNA-mRNA association (3, 5, 4). This observation suggests that RNA accessibility for intermolecular interactions depends on their intramolecular folding: regions engaged in stable intramolecular base-pairing are sequestered and unavailable for interactions with other RNAs, while unstructured regions remain accessible. We therefore hypothesized that the extent of mRNA-mRNA interactions in *E. coli* is set by the balance between folded (sequestered) and unstructured (accessible) sequence.

**Figure 1.**
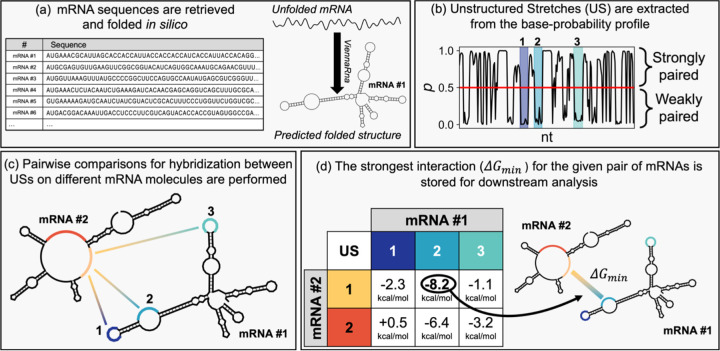
Pipeline for predicting mRNA intermolecular interactions. (a) All annotated mRNA sequences are retrieved from a transcriptomic compendium (22) and folded using ViennaRNA (57). (b) The regions corresponding to long stretches of nucleotides (≥ 7 nt) having low base-pairing probability (*p* < 0.5) are annotated as Unstructured Stretches and highlighted as shaded area in the graph. In the example for mRNA #1, three USs satisfy our criteria. (c) For all pairs of mRNAs in the dataset, the free energy for hybridization for all their USs is calculated. In the example, mRNA #1, having three USs, is compared with mRNA #2, having 2 USs. (d) The most stable interaction per pair of mRNAs is stored for the simulation. In the example, the largest change in free energy is observed when the US number 2 of mRNA #1 hybridizes with the US number 1 of mRNA #2.

**Figure 2.**
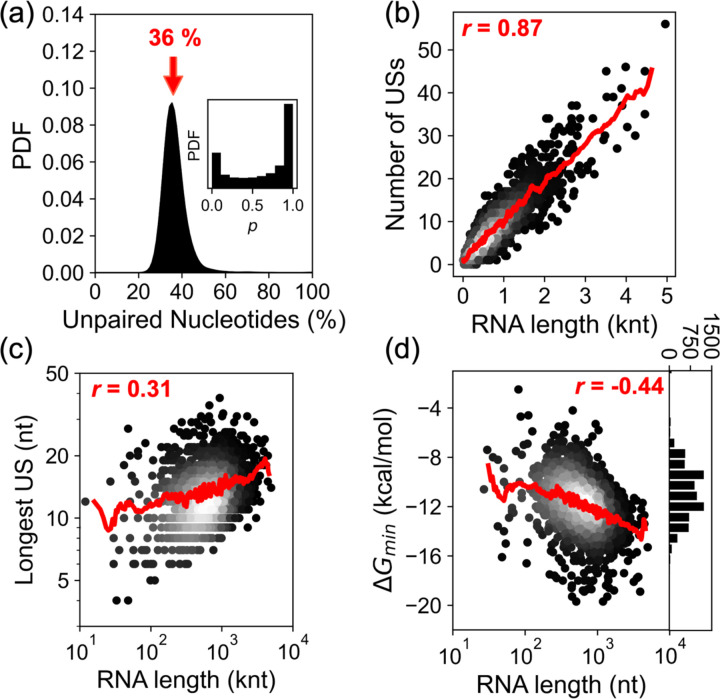
Accessibility and interaction potential across *E. coli* mRNAs. (a) Fraction of unpaired nucleotides per transcript; arrow marks the median (36%). Inset shows distribution of pairing probabilities in our dataset. (b) The number of unstructured stretches (length ≥ 7 nts) per transcript versus transcript length (knt = 10^3^ nts); Pearson’s r = 0.87. (c) The length of the longest unstructured stretch versus the transcript length; Pearson’s r = 0.31. (d) The hybridization energy for the strongest hybridizing stretch (*ΔG*_*min*_) of a given transcript versus its length; Pearson’s r = −0.44. Red lines show moving averages. Histogram shows distribution of values for the strongest *ΔG*_*min*_ per transcript.

To identify these accessible regions, we folded each mRNA individually, inherently favoring intramolecular base-pairing, then identified the remaining unstructured sequences as candidates for trans interactions (see Methods A, Fig. 1). Each mRNA sequence was folded *in silico* using ViennaRNA to predict its intramolecular secondary structure, and nucleotides with low pairing probability (our “weakly paired” criterion; see Equation (2), Methods B) were extracted. This analysis revealed that across the transcriptome, ~36% of mRNA nucleotides are unlikely to be paired under our threshold and thus potentially accessible for intermolecular interactions (Fig. 2(a)).

We next extracted USs consisting of at least 7 continuously “weakly paired” nucleotides. Notably, we excluded stretches interrupted by even one or two strongly paired bases. The number of unstructured stretches per transcript scaled strongly with the transcript length (Pearson’s Correlation Coefficient *r* = 0.87; Fig. 2(b)), with a median density of ~10 USs per 1,000 nucleotides. The length of the longest unstructured stretch also showed a modest positive correlation with transcript length (Pearson’s Correlation Coefficient *r* = 0.31, log-log space; Fig. 2(c)), indicating that longer mRNAs harbor both more and longer single-stranded segments.

With this approach, we annotated ~46,000 USs across 4,510 transcripts. In the steady-state pool (7,569 mRNAs/cell), this corresponds to ~55,000 USs total with a median length of 8 nucleotides. The likelihood of hybridization among these putative USs would depend on the availability of complementary sites. To evaluate the potential for intermolecular RNA-RNA interactions, we performed exhaustive pairwise comparisons across all unstructured stretches (see Methods C). Each unstructured stretch was treated as a potential interaction interface, and for each mRNA pair, we identified the most stable interface, and recorded the hybridization energy, *ΔG*_*min*_, for downstream analysis.

This analysis produced a matrix of ~29 million potential interactions (the upper triangle of a 7,569 × 7,569 matrix). The strongest *ΔG*_*min*_ for each transcript became increasingly negative with transcript length (Pearson’s correlation coefficient *r* = −0.44, semi-log scale; Fig. 2(d)), consistent with longer transcripts harboring longer unstructured segments. Across the 7569 sequences, the median value of the strongest hybridization energy was −11 kcal/mol, comparable to the stability of an 8-bp RNA duplex, such as those formed by miRNA seed regions (23). The most stable interaction observed in our system was −20 kcal/mol, corresponding to a duplex of roughly 13 bp in length, approaching the binding energy of therapeutic antisense oligonucleotides (24) (Fig. 2(d)).

Out of the ~29 million pairs of RNAs in our system, roughly 28 million registered valid interactions with negative *ΔG*_*min*_, and ~2 million meet the criterion for “strong” interactions, defined by an empirical threshold of *ΔG*_*min*_ ≤ −7 kcal/mol (~ −12 kT). After filtering for these qualifying interactions, ~38,000 unique USs remained capable of strong binding, averaging 5 “strong” interaction patches per RNA. Employing a simple indefinite self-association model (see SI 1 for details) for a system containing 20 μM RNA with −7 kcal/mol interaction patches, we found that a remarkable ~70% of the molecules is expected to be part of aggregates at equilibrium (25).

Because ~2 million “strong” interactions are theoretically possible in the system, but only ~38 thousand unique USs are present, these cannot all be realized simultaneously. More realistically, each unstructured stretch can interact with only one among many multiple partners at a given time, leading to competition for binding sites that can create many alternative configurations of the system. To capture this competition and turnover that cannot be described with a simple self-association model, we implemented kinetic stochastic simulations that evolve US-US binding and unbinding over time and report mRNA-level outcomes.

### 2. Stochastic simulation of transcriptome-wide interactions

To determine if the high potential for static interactions translates into dynamic aggregation, we performed kinetic stochastic simulations of US-US binding and unbinding across the entire copy-number weighted *E. coli* transcriptome. The simulation follows a scheme based on the Gillespie algorithm (26), with thermodynamically-grounded reaction rates (see Methods D). Reported hybridization rates (*k*_*on*_) for nucleic acids in literature span between 10^6^ to 10^7^ M^−1^s^−1^(27, 28, 17). Although length, CG content (17), coaxial stacking (29) and defects (mismatches, bulges) (30) influence the hybridization rate, their effect on *k*_*on*_ is typically modest when compared to their impact on the dehybridization rate (*k*_*off*_). We therefore fixed *k*_*on*_ = 10^7^ M^−1^s^−1^ in line with the values reported for RNA oligonucleotides (17). The specific dehybridization rate *k*_*off*_ for each US-US pair was computed from the hybridization free energy, using Equation (3). This analysis yielded dissociation timescales that could be as long as ~2 years for the most stable duplexes in our set.

Because the Gillespie algorithm tracks individual molecules, we converted the concentration-dependent hybridization rate *k*_*on*_ to an effective per-pair reaction rate. Given the *E. coli* intracellular volume of 6.7 × 10^−10^ μL (21), the effective concentration of a single molecule is approximately 2.5 nM, yielding a constant hybridization rate of 2.5 × 10^−2^ s^−1^ for any pair of available unstructured stretches.

The simulation proceeds one event at a time, consisting of either the hybridization of two unstructured stretches on two mRNAs to form a new duplex, or the dehybridization and dissolution of an existing one. Since low hybridization energies correspond to fast dynamics, weak duplexes form and dissolve at high frequency. Including these events would force the simulation to spend the vast majority of its time processing these transient interactions. In this case, simulating all possible pairwise interactions would be computationally prohibitive. To overcome this, we introduced a thermodynamic cut-off and focused the simulation only on the more stable interactions, having *ΔG*_*min*_ ≤ −7 kcal/mol (see SI 2 for the evaluation of different thresholds). This practical compromise ensured computational feasibility while retaining the most stable and likely consequential interactions.

### 3. Simulations predict widespread aggregation of the E. coli transcriptome

**Figure 3.**
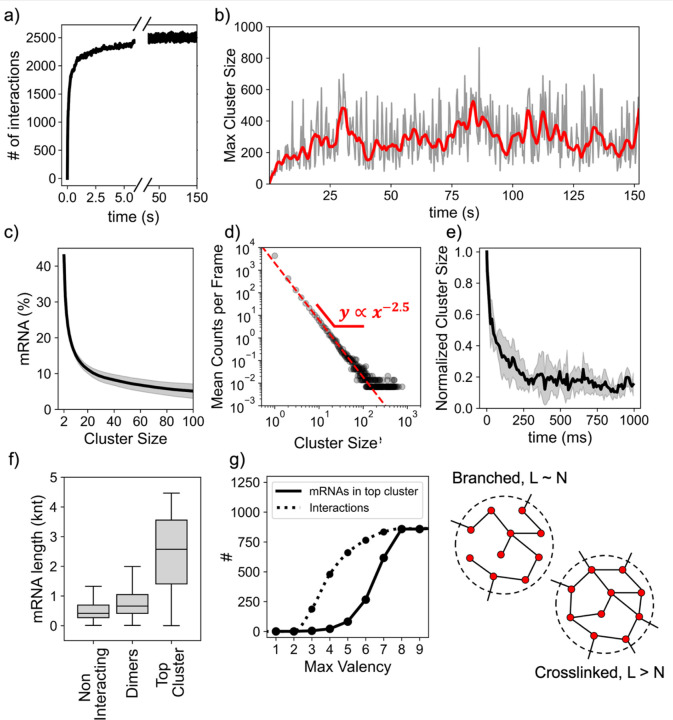
Kinetic simulations of mRNA interactions in *E.coli* show widespread aggregation. (a) Simulated experiment shows the rapid formation of mRNA-mRNA intermolecular interactions and the establishment of a steady state counting ~2500 transient interactions. (b) Size of the largest cluster fluctuates on fast timescales, with clusters comprising as many as ~900 individual mRNAs assembling and disassembling dynamically over time. Bold red line shows moving average. (c) Distribution of mRNA in aggregates of size ≥ Clusters Size at steady state. The clusters in the simulation dynamically form and break apart, but at steady state, on average ~ 40% of the total population is interacting, and ~15% of the total population is part of clusters having size ≥ 10. (d) The distribution of clusters sizes at steady state follows a power law with a strong scaling exponent of −2.5. (e) Interactions between RNA in the simulation are short-lived, with large clusters dissolving in less than a second. The trace shows the normalized mean trajectory of five clusters with initial size ≳ 700. (f) The largest cluster is heavily enriched in long mRNAs. Boxplots of mRNA length for non-interacting RNA monomers, dimers, and members of the top cluster (g) Removal of high valency molecules causes the top cluster to break apart, showing that high valency mRNAs behave as hubs that hold small assemblies together, and revealing an underlying branched topology. Sketches represent the topology of a branched cluster and a crosslinked cluster having the same number of molecules (N) but different number of interactions (L).

Our kinetic simulations revealed that the transcriptome is intrinsically poised to form a dynamic network of aggregates. The system rapidly approaches a steady state, with approximately 2,500 interactions (Fig. 3(a)), where ~40% of all mRNA molecules are engaged in intermolecular base-pairing, with frequent hybridization and dehybridization events.

Because each transcript can have multiple USs, individual transcripts can bind several partners. This intrinsic multivalency results in the spontaneous formation of large RNA assemblies (Fig. 3(b)). At steady state, ~15% of all mRNA molecules are part of clusters containing ten or more molecules, and ~10% of all mRNA molecules are part of clusters of 20 or more molecules (Fig. 3(c)). The cluster-size distribution followed a power law with an exponent of −2.5 (Fig. 3(d)), consistent with mean-field gelation as described by Flory–Stockmayer theory(31). This strong scaling means that most interactions lead to dimers or small oligomers, but a significant fraction of transcripts coalesce into large assemblies, with the largest cluster observed in our simulations containing as many as ~900 molecules.

Remarkably, these assemblies were highly dynamic, with simulations showing fast fluctuations and large jumps in the size of the largest cluster from frame to frame. By tracking their dynamics at high resolution, we found that very large clusters (size ≳ 700) typically lost ~80% of their elements within ~60 ms after reaching a local maximum size (Fig. 3(d)). This rapid turnover arises from the extremely short median lifetime of mRNA–mRNA interactions (1/*k*_*off*_) of only a few seconds in both dimers and large clusters. As a result, even the largest clusters persist only for a fraction of a second before reorganizing, as individual transcripts bind and unbind.

Further analyzing the composition of our aggregates, we found that the largest clusters were heavily enriched in long transcripts. mRNAs in the largest aggregates had a median length of ~2,500 nucleotides compared to ~700 nucleotides in dimers and ~400 nucleotides for unbound mRNAs (Fig. 3(f)). This bias mirrors the expectation from Fig. 2 (longer transcripts have more/longer USs), and mirrors the experimental observations that long RNAs are preferentially recruited to stress granules (3).

Interestingly, in these assemblies, the total number of interactions (L) is comparable to the total number of molecules (N), indicative of branched networks lacking extensive crosslinks. Within large aggregates, most molecules were engaged with only a small number of partners at the same time. We found that the number of interactions follows an approximately exponential distribution, resulting in relatively few highly connected species.

This topology suggested that our large assemblies could effectively be described as collections of small clusters held together by multivalent hubs. To test this hypothesis, we selectively removed molecules with high valency from the largest cluster (Fig. 3(g)). We found that limiting the maximum valency to 6 by removing molecules with higher connectivity from the system, caused the loss of only ~100 total interactions. If these molecules were at the edges of the cluster or within a highly crosslinked network, we would have expected removing these molecules (accounting for ~4% of all contacts) would yield only a modest decrease in cluster size. Instead, we found that this process led to fragmentation of the main cluster into smaller assemblies, the larger of which contained only ~270 molecules. This observation indicates that these multivalent elements behave like hubs, joining smaller clusters together. These hubs are typically long transcripts that are rich in USs allowing higher degrees of connectivity (Fig. 2). For example, *gltB*, one of the longest *E. coli* transcripts, was found to be engaged in up to nine simultaneous interactions.

Together, these simulations reveal that in the absence of translation, RBPs, and other cellular countermeasures, the sequence-specific properties of mRNAs poise the transcriptome toward widespread aggregation. Notably, our model employs conservative estimates and RNA’s aggregation propensity in the cellular environment may be even stronger than our simulations indicate. To independently validate that mRNAs indeed possess this inherent tendency toward aggregation, we next tested whether natural selection has left signatures in mRNA sequences by minimizing their intermolecular interactions.

### 4. mRNAs are evolutionarily selected to minimize intermolecular interactions

Since aggregation can interfere with translation and key cellular processes, we hypothesized that natural selection may have shaped mRNA sequences to minimize intermolecular hybridization. To test this, we compared the properties of the native *E. coli* transcriptome against a carefully designed null model consisting of dinucleotide-preserving shuffles which maintain the same potential for base-pairing but lack any evolved, sequence-specific information. We found three distinct lines of evidence for this selective pressure.

**Figure 4.**
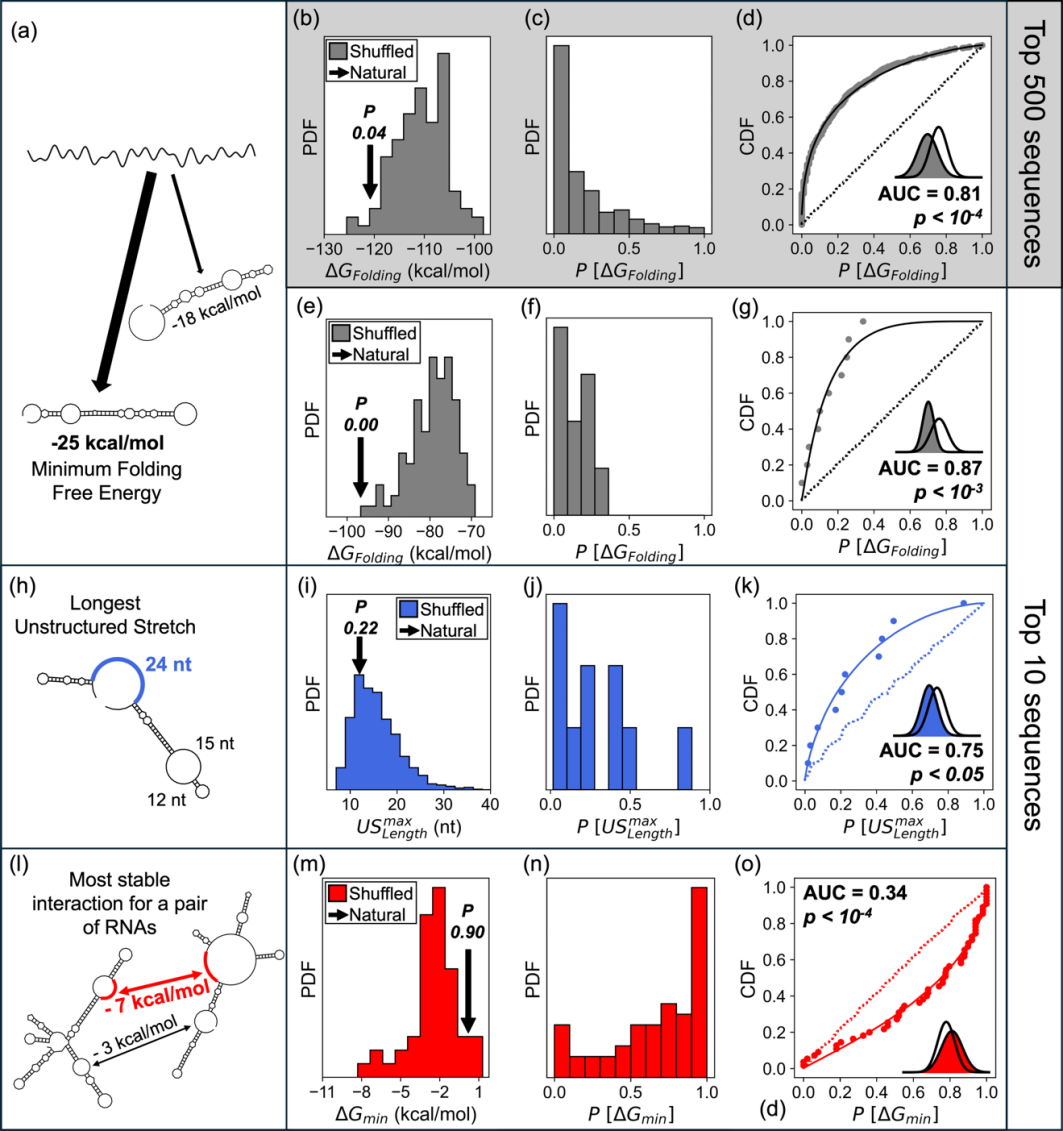
Native *E. coli* mRNAs fold better, expose shorter USs, and interact more weakly than sequence-shuffled controls. (a-d) Analysis of folding for the 500 most abundant mRNAs in *E. coli*. (a) The minimum free energy for the folding of a naturally occurring sequence is calculated. (b) The folding energy is compared against shuffled sequences as illustrated for a representative case. Native sequence is more stable (indicated by arrow). (c) The distribution of probabilities for the naturally occurring sequences to fold more weakly than their randomized counterpart is shown, highlighting how *P*[*ΔG*_*Folding*_] tends to zero. (d) The AUC of the empirically determined CDF for *P*[*ΔG*_*Folding*_]. The dotted line traces the calculated CDF for the null-distribution of *P* values, and the continuous lines show the expected CDF for the one-tail probabilities of sampling values from a normal distribution that are as negative as values sampled from a Gaussian distribution with mean −1.3 *δ*. The inset depicts the equivalent shift for a Gaussian distribution. (e-g) Same analysis as (b-d) for the 10 most abundant mRNAs in *E. coli* showing a stronger effect among these abundant transcripts. (h,i, j, k) Analysis of the longest USs in the 10 most abundant mRNAs in *E. coli*. (h) Sketch showing how $US_{\text{Length}}^{\text{max}}$ is computed for a given sequence. The values for the naturally occurring USs are compared against shuffled sequences as depicted in (i) for a representative case, showing how it is unlikely to sample USs that are shorter than the naturally occurring ones among the 10 most abundant mRNAs. (j) The distribution of probabilities that a native transcript’s longest unstructured stretch is at least as long as in shuffled controls $P\left[US_{\text{Length}}^{\text{max}}\right]$; the skew toward 0 indicates native sequences generally have shorter longest USs. (k) The AUC of the empirical CDF for all the $P\left[US_{\text{Length}}^{\text{max}}\right]$ values is skewed to low *P* values. The dotted line shows the calculated AUC for the null-distribution of *P* values, and the continuous lines show the expected CDF for the one-tail probabilities of sampling values from a normal distribution that are as negative as values sampled from a Gaussian distribution with mean +0.9 *δ*. The inset depicts the equivalent shift for a Gaussian distribution. (l,m,n,o). Analysis of the interaction energies between the 10 most abundant mRNAs in *E. coli*. (l) Sketch showing how *ΔG*_*min*_ is computed between a pair of sequences. The values for the naturally occurring pairs are compared against shuffled sequences as depicted in (m) for a representative case, showing how it is likely to sample interaction energies that are smaller than the naturally occurring ones among the 10 most abundant mRNAs. (n) The distribution of probabilities for the naturally occurring sequences to interact more weakly than their randomized counterpart, highlighting how *P*[*ΔG*_*min*_] tends to one. (o) The AUC of the empirically determined CDF for all the *P*[*ΔG*_*min*_] values is skewed to high *P* values. The dotted line shows the calculated AUC for the null-distribution of *P* values, and the continuous lines show the expected CDF for the one-tail probabilities of sampling values from a normal distribution that are as negative as values sampled from a Gaussian distribution with mean +0.7 *δ*. The inset depicts the equivalent shift for a Gaussian distribution.

We first tested whether mRNA sequences have been selected to optimize intramolecular folding. We reasoned that more stable intramolecular folding would minimize USs and thus reduce intermolecular interactions. We examined the top 500 most abundant mRNAs in *E. coli*. For each mRNA sequence, we generated 100 dinucleotide shuffled sequences using the Altschul-Erikson algorithm (32), that keeps length and dinucleotide composition constant while randomizing higher-order sequence features. This approach conserves nearest-neighbor stacking propensities (10), and thus isolates the contribution of sequence-specific information to RNA folding beyond simple compositional effects. We then computed the minimum free energy of folding (*ΔG*_*Folding*_) for each shuffled sequence (Fig. 4(a)).

To avoid assumptions on the underlying distribution of the folding energies, we employed a non-parametric approach. For each sequence, we computed the one-tail probability (*P*[*ΔG*_*Folding*_]) of obtaining a folding energy as negative as the natural sequence from its corresponding null distribution (Fig. 4(b)), and summarized the ensemble by the empirical CDF of *P*-values and its AUC (Methods E, Fig. 4(c, d). Strikingly, natural sequences showed significantly more stable folding than their shuffled counterparts (AUC = 0.81, *P*-value < 10^−4^), with an effect size on the *ΔG*_*Folding*_ energies equivalent to shifting a normal distribution by −1.3 standard deviations (*δ*). This trend was even more pronounced for the ten most abundant transcripts (AUC = 0.87, *P*-value < 10^−3^, effect size ≈ −1.3 *δ*), which are at the highest risk of aggregation due to their concentration. These results indicate a strong selective pressure for mRNAs to adopt stable structures.

Next, we examined whether this stable intramolecular folding reduces unstructured regions, the primary drivers of aggregation. We focused on the ten most abundant mRNAs where evolutionary pressure would be most acute. For each of these mRNAs, we measured the length of the longest US ($US_{\text{Length}}^{\text{max}}$, Fig. 4(h)) in both the native sequence and dinucleotide shuffled versions. Our analysis revealed that native sequences harbor significantly shorter maximal USs than their randomized counterparts (AUC = 0.75, P < 0.05) (Fig. 4(i–k)). The effect on the $US_{\text{Length}}^{\text{max}}$ values was comparable in magnitude to shifting a normal distribution by −0.9 *δ*. For 9 out of 10 sequences, the natural version had shorter maximum US lengths than the majority of shuffled variants. These results indicate that evolution has minimized these potential “sticky patches” in the most abundant transcripts.

Finally, we examined whether these structural adaptations translate to functional consequences and affect the strength of intermolecular mRNA interactions. For each pair among the 10 most abundant mRNAs (55 unique pairs accounting for homotypic pairs), we generated 50 pairs of dinucleotide shuffled mRNA sequences. For every native or shuffled pair, we enumerated all candidate US-US alignments between the two transcripts, computed duplex free energies, and recorded the most stable value (*ΔG*_*min*_). For each native pair we then assigned a one-tailed, non-parametric *P*-value and summarized evidence across pairs by the *P*-value CDF AUC as described above. Strikingly, natural mRNA pairs consistently formed weaker interactions than their randomized counterparts (AUC = 0.34, p < 0.001; Fig. 4(m–o)), with an effect size corresponding to a normal-shift equivalent of + 0.7 *δ*. For 41 out of 55 pairs, the native sequences formed weaker interactions than the majority of shuffled versions. Thus, among the most abundant transcripts, random rearrangement of the same dinucleotides would create substantially stronger, inter-molecular interactions than the native sequence.

Together, these three lines of evidence: enhanced intramolecular folding, shortened unstructured regions, and weakened intermolecular interactions, demonstrate that *E. coli* mRNA sequences have evolved to minimize promiscuous intermolecular pairing, thereby attenuating the intrinsic aggregation propensity revealed by our simulations.

## Discussion

Our study combines large-scale simulation with evolutionary analysis to reveal a fundamental, yet overlooked, property of the transcriptome: its intrinsic propensity to aggregate. By modeling the entire *E. coli* transcriptome in the absence of proteins and translation, we show that the physicochemical properties of RNA alone are sufficient to drive the formation of a dynamic network of intermolecular contacts. At steady state, ~40% of mRNA molecules in our simulations are expected to take part in intermolecular interactions, with base-pairing between unpaired stretches leading to widespread aggregation. Crucially, we find that this inherent aggregation propensity is strong enough to constitute a selection pressure on genome sequences.

Our model predicts extensive RNA aggregation, yet this is not observed in unperturbed cells, implying that cells must possess potent mechanisms to counteract this fundamental physical drive. Factors like ribosome occupancy, helicases, and RBPs likely shield accessible regions or actively disrupt promiscuous interactions. Our RNA-only baseline therefore provides a quantitative reference for assessing both the extent of evolutionary selection against aggregation and the efficiency of these cellular mechanisms. For example, our framework enables estimation of the minimum ribosome coverage or RBP occupancy required, beyond sequence optimization, to maintain dispersed mRNAs *in vivo*.

Among the outcomes of our simulations, we found that longer transcripts exhibit higher valency and drive the formation of larger RNA clusters: a pattern similar to experimental observations from stress granules in eukaryotic cells that form upon acute translation inhibition (3). Consistent with this, protein-free, purified mRNA self-assembles into clusters that recapitulate stress granule composition (3). Our simulations suggest that these longer RNAs may act as multivalent hubs that connect smaller clusters into larger assemblies. Rather than simply preventing aggregation, cells appear to harness this fundamental property of RNA for functional organization. Many cellular condensates require RNA for their formation (33), suggesting that controlled RNA aggregation may provide a foundation for cellular compartmentalization. Cells may have even evolved specialized scaffolding RNAs to template condensates. By strategically placing interaction sites, these RNAs could turn inter-molecular RNA interactions into a programmable organizational tool.

Most notably, our findings reveal previously unrecognized selection pressures that have shaped genomic sequences to counteract RNA aggregation. We demonstrate that native *E. coli* mRNAs are substantially more structured than one would expect just by chance. This hypothesis previously found support by Seffens and colleagues (34) and was later contested by Workman (35) and Clote (32). We resolve these seemingly conflicting reports by using a larger, organism-specific dataset with rigorous statistical controls. Besides enhanced intramolecular folding, we show that the most abundant mRNAs have evolved shorter unstructured regions and weaker intermolecular contacts than their dinucleotide-shuffled counterparts. These observations suggest the existence of avoidance mechanisms between mRNA molecules, similar to what has been previously observed for abundant non-coding RNAs (19, 36). Moreover, our results suggest that beyond optimizing codon usage and tRNA abundances, natural selection has systematically fine-tuned mRNA sequences to maintain transcriptome solubility, and this fundamental constraint may have broadly influenced genome evolution across organisms.

Furthermore, our work establishes a physical principle that yields testable predictions. The baseline aggregation pressure is a direct function of mRNA concentration. It should therefore increase during rapid growth (when mRNA copy numbers are highest) and under stresses that cause polysome disassembly, which unmasks stretches of previously protected RNA (37). Cells may also need to contend with conditions that directly promote RNA-RNA interactions. For instance, cold stress may thermodynamically stabilize spurious intermolecular base pairs, increasing aggregation risk. In response, cells dramatically upregulate cold shock proteins (e.g., CspA in *E. coli*), specialized RNA chaperones/helicases that resolve inappropriate duplexes and maintain solubility (38). The very existence of this sophisticated, inducible system serves as strong evidence for the selective pressure described in our work. Other stresses, such as intracellular pH shifts or osmotic shock (39, 40), could similarly promote aggregation, highlighting that maintaining transcriptome solubility requires constant adaptation to physiological and environmental change.

While our simulations provide insight into RNA aggregation propensity, it is important to consider that nucleic acid biophysics inside living cells may differ fundamentally from expectations based on studies in buffered solutions. Most nucleic acid thermodynamics and kinetics studies have been performed under dilute *in vitro* conditions on short oligonucleotides, typically neglecting the complex chemical composition of the intracellular milieu and potential deviations due to the large size of native transcripts (41). Notably, up to 40% of the cytosol is occupied by macromolecules (42). Recent studies have examined the contribution of molecular crowders (43–45) and reconstituted *E. coli* cytoplasm (46) on RNA folding, finding measurable but modest differences from dilute buffer conditions. Large scale chemical probing studies in yeast and mammalian cells have shown that RNA molecules *in vivo* can be less structured than *in vitro*, though the mechanisms and magnitude of these differences remain unclear (47, 48). While these factors may reduce aggregation in cells, the effects observed so far appear insufficient to fully explain the striking absence of widespread RNA aggregation *in vivo*. This gap between our predictions and cellular reality underscores either the existence of additional active regulatory mechanisms or more fundamental differences in cellular RNA thermodynamics that remain to be discovered (49).

Regardless of the precise mechanisms, our work provides a new lens for understanding RNA biology and regulation. By revealing the transcriptome’s inherent aggregation potential and the evolutionary adaptations that counteract it, we establish a framework for investigating how cells manage their RNA pools. This perspective suggests that many cellular processes, such as co-translational folding (50), continuous surveillance by RBPs (51), and the inherently short half-life of mRNAs, may be shaped in part by the need to maintain transcriptome solubility. Understanding these constraints and solutions will be essential for deciphering principles of cellular organization and may reveal new therapeutic targets for diseases involving RNA aggregation and dysregulated biomolecular condensates.

## Materials and Methods

### A. Sequencing data and RNA abundance

To study the *E. coli* transcriptome, we used the K-12 MG1655 genome (assembly ASM584v2) obtained from RefSeq (52). RNA sequences were taken from the recently published compendium from Tjaden (22) (available in the Harvard Dataverse at https://doi.org/10.7910/DVN/QBMC9D, Assemblies Table 2) and ncRNA, rRNA and tRNA were filtered out. For each mRNA, a median transcript abundance was computed and stored in an array *A*.

Assuming 7,800 mRNA molecules in a single *E. coli* cell grown in LB (20) we converted the copy number for the *i*-th mRNA based on its transcript abundance as:

(1) $$N_{i}=\left\lfloor \frac{A_{i}*7800\frac{\text{transcript}}{\text{cell}}}{\sum A}\right\rfloor$$

This yielded a total of 7,569 RNA molecules after rounding down.

### B. RNA folding *in silico*

To determine accessible regions along the transcripts that are potentially available for base-pairing, the RNA sequences were folded *in silico* using the ViennaRNA 2.6.4 Python library on the Whitehead Institute BaRC Fry cluster. Instead of relying on the minimum free energy (MFE) structure, we opted for a more conservative approach previously established for the prediction of antisense oligonucleotides binding sites (53), where every nucleotide *i* over one mRNA of length *L* is considered as “weakly paired” when:

(2) $$P_{i}=\sum_{j=1}^{L}p_{i,j}<0.5$$

where *p*_*i,j*_ is the base-pair probability for bases *i* and *j*. When compared to directly using the centroid structure of the Boltzmann ensemble (54), our approach is more stringent, although as a downside it does not necessarily return a single physically meaningful fold. Because *in silico* predictions tend to overestimate structure (47, 48, 55), this approach likely underrepresents the true number of accessible regions and serves as a conservative estimate of interaction potential (see SI 3 for evaluation of ViennaRNA performance).

### C. Prediction of interactions among *E. coli* mRNAs

To evaluate potential interactions among mRNAs, we first retrieved single stranded regions along the transcripts. To do so, for each mRNA, we extracted stretches having at least 7 adjacent “weakly paired” nucleotides (nts). The 7-nt threshold was chosen to avoid introducing an excessively large number of extremely short stretches that would have made computations unfeasible. Importantly, in this work we disregarded the role of RNA strand displacement to be more conservative (14, 17), and discarded stretches that would have been 7 or more nt-long if they were not interrupted by just one or two “strongly paired” nucleotides.

For every pair of transcripts, we computed the MFE for hybridization between any of their annotated unstructured stretches using ViennaRNA *duplexfold* function. A triangular (7569 × 7569) interaction matrix was filled at any *i,j* entry by storing the most stable hybridization energy among all possible hybridizing stretches for any two transcripts *i* ad *j* (*ΔG*_*min*(*i,j*)_). For each entry of the matrix, a corresponding equilibrium constant was calculated:

(3) $$K_{i,j}=c^{⊖}e^{\frac{\Delta G_{\text{min}\left(i,j\right)}}{\text{RT}}}=\frac{k_{\text{off}}}{k_{\text{on}}}$$

with T being the absolute temperature (310.15 K), R the gas constant (1.987 × 10^−3^ kcal/mol/K) and c^⊖^ the standard reference concentration (1 mol/L).

### D. Kinetic stochastic simulations

To evaluate the RNA interaction potential inside an *E. coli* cell, we set up a custom kinetic stochastic simulation. The code was written in Python and accelerated using just-in-time (JIT) compilation provided by the Numba library (56), and executed on the Whitehead Institute BaRC Fry cluster.

The simulation strategy was based on the Gillespie algorithm for the simulation of networks of chemical reactions (26). Briefly, at each time point *t* of the simulation, the state of the system is defined by the comprehensive list of all RNA molecules and their state (paired/unpaired). From this list, we compute “on the fly” all possible reactions *i ∊ χ* that can occur in the current state, and their respective rates *r*_*i*_. To evolve the system, we (i) sample the waiting time *τ* for the next reaction from the exponential distribution with mean equal to (∑_*i∊χ*_ *r*_*i*_)^−1^ to update *t → t + τ*, and (ii) randomly select a reaction by sampling with probability proportional to *r*_*i*_/∑_*i∊χ*_ *r*_*i*_. The two steps are repeated until the simulation reaches the chosen time limit, and the state of the system is exported at fixed intervals for further analysis.

### E. Calculating AUCs and *p*-values

To test whether *E. coli* sequences were significantly different from a pool of shuffled sequences, we used a non-parametric approach. Briefly, for each examined metric (e.g., folding free energy) we computed the one-tail probability of obtaining the metric value as or more extreme as the naturally occurring one, when sampling from the null-distribution obtained from shuffled sequences. This approach converts the metric values to *P* values. We then quantified the overall deviation from the null by plotting the empirical Cumulative Distribution Function (CDF) of these P-values and computing its Area Under the Curve (AUC). The AUC is extremely sensitive to changes in distributions of *P*-values. Under the null, P-values are uniformly distributed, and AUC is expected to be equal to 0.5. A distribution skewed towards low *P* values yields AUC > 0.5, and a distribution skewed towards large *P* value will result in AUC < 0.5.

We further assessed the significance of the observed AUC by bootstrapping. We generated 10^4^ synthetic datasets with same structure as the original one, by sampling from the pool of randomized sequences. For each synthetic dataset we computed the AUC, and evaluated the one-tail probability of observing a sample with an AUC at least as extreme as the one determined for naturally occurring sequences, yielding a bootstrap *p*-value for the observed AUC.

To provide an intuitive measure for the magnitude of the difference between the naturally occurring sequences and their randomized counterparts, we computed the equivalent shift for a normal distribution that would yield the same AUC. Briefly, we considered that the values of a metric of interest for naturally occurring sequences were part of a distribution that was effectively shifted when compared to the shuffled pool of values (null distribution). We determined the one-tail probability of sampling values from a normal distribution that were at least as extreme as values sampled from a normal distribution that was shifted by some multiple of a standard deviation (*δ*) and potentially having a different variance. The combination of shifts (*δ*) and changes in variance that generated CDFs reproducing the CDFs of the original metrics of interest were annotated, and their effective shifts were reported.

## Supplementary Material

Supplement 1

## Significance Statement

RNA molecules are not inert carriers of genetic information: their chemistry can drive collective behaviors in cells. We developed a first-principles computational framework to quantify how much the transcriptome would aggregate if governed solely by sequence-encoded interactions in a cellular volume. Our simulations reveal that hybridization energies are sufficient to drive extensive mRNA aggregation into dynamic networks, with long transcripts acting as multivalent hubs—paralleling the organization of stress granules. Complementary evolutionary analyses show that native mRNAs bear signatures of selection against aggregation, favoring intramolecular folding to limit promiscuous intermolecular binding. These findings illuminate how RNA physical chemistry has constrained genome evolution and provide a new lens to interpret RNA behavior inside the cell.
