# Supplementary material for: Transcriptome-wide analysis reveals sequence selection to avoid mRNA aggregation in E. coli
Source: bioRxiv. 2025 Sep 20:2025.09.19.677370. Preprint. [Version 1] doi: 10.1101/2025.09.19.677370 (PMC12458354; doi:10.1101/2025.09.19.677370)
Supplement: Supplement 1 [file media-1.docx]

**Supporting Information for**

Transcriptome-wide analysis reveals sequence selection to avoid mRNA aggregation in *E. coli*

Marco Todisco^1^ and Ankur Jain^1,2,*^

^1^Whitehead Institute for Biomedical Research, Cambridge, MA, 02142, USA

^2^Department of Biology, Massachusetts Institute of Technology, Cambridge, MA, 02139, USA.

*Corresponding author.

**Email:**  ajain@wi.mit.edu

**This PDF file includes:**

Supporting text

Figures S1 to S2

SI References

Supporting Information Text

1. **Indefinite self-association model**

To estimate the interacting fraction in a system composed by self-associating molecules, we have implemented a simple indefinite self-association model (equal *K*) as described by Martin (1).

Briefly, for a system of self-associating RNA molecules, it is possible to write a set of successive equilibria and associated equilibrium constants:

$\left[ RNA \right]+\left[ RNA \right]\rightleftharpoons{[RNA]}_{dimer}\Longrightarrow K_{dimer}=\frac{\left[ RNA \right]^{2}}{\left[ RNA \right]_{dimer}}=c^{⊖}e^{\frac{\Delta G}{RT}}$

${[RNA]}_{dimer}+\left[ RNA \right]\rightleftharpoons{[RNA]}_{trimer}\Longrightarrow K_{trimer}=\frac{[RNA]\left[ RNA \right]_{dimer}}{\left[ RNA \right]_{trimer}}=\frac{\left[ RNA \right]^{3}}{K_{dimer}\left[ RNA \right]_{trimer}}$

${[RNA]}_{trimer}+\left[ RNA \right]\rightleftharpoons{[RNA]}_{tetramer}\Longrightarrow K_{tetramer}=\frac{[RNA]\left[ RNA \right]_{trimer}}{\left[ RNA \right]_{tetramer}}=\frac{\left[ RNA \right]^{4}}{K_{dimer}K_{trimer}\left[ RNA \right]_{tetramer}}$

And so on to infinite *n*-mer. The total molar concentration of [RNA] would then be given by:

$${[RNA]}_{tot}=\left[ RNA \right]+2{[RNA]}_{dimer}+3{[RNA]}_{trimer}+4{[RNA]}_{tetramer}\ldots$$

Which can be rewritten as:

$${[RNA]}_{tot}=\left[ RNA \right]\left( 1+\frac{2[RNA]}{K_{dimer}}+\frac{3\left[ RNA \right]^{2}}{{K_{dimer}K}_{trimer}}+\frac{{4\left[ RNA \right]}^{3}}{{{K_{dimer}K}_{trimer}K}_{tetramer}}+\ldots\right)$$

Under the simplifying assumption that all dissociation constants are equal, so that growing an aggregate does not introduce any bonus nor penalty, we can define some useful constants:

$$x=\frac{[RNA]}{K}$$

$$L=\frac{{[RNA]}_{tot}}{K}$$

And rewrite $L$ as follows:

$$L=x\left( 1+2x+3x^{2}+4x^{3}+\ldots\right)$$

Since in such self-aggregating systems we find that *x* < 1, the series in parenthesis can be solved, obtaining the following:

$$L=\frac{x}{\left( 1-x \right)^{2}}$$

Which can be expanded as a quadratic equation in $x$, yielding the positive solution:

$$x=\frac{2L+1-\sqrt{4L+1}}{2L}$$

Finally, the fraction of monomers can be written as:

$$\frac{[RNA]}{{[RNA]}_{tot}}=\frac{x}{L}=\frac{2L+1-\sqrt{4L+1}}{2L^{2}}$$

By setting ${[RNA]}_{tot}=20 \mu M$ and $\Delta G=-7 kcal/mol$, the expected aggregated fraction ($\theta$) inside the cell can easily be computed as:

$$\theta=1-\frac{2L+1-\sqrt{4L+1}}{2L^{2}}=1-\frac{\frac{2\left[ RNA \right]_{tot}}{c^{⊖}e^{\frac{\Delta G}{RT}}}+1-\sqrt{\frac{4\left[ RNA \right]_{tot}}{c^{⊖}e^{\frac{\Delta G}{RT}}}+1}}{2\left( \frac{\left[ RNA \right]_{tot}}{c^{⊖}e^{\frac{\Delta G}{RT}}} \right)^{2}}\approx0.7$$

1. **Energy thresholds**

When performing our analysis of the E. coli transcriptome, we retrieved ~70,000 hybridizing stretches with energies equal or lower than -10 kcal/mol. For two molecules at 2.5 nM, this binding energy would correspond to a binding probability at equilibrium roughly larger than 3%. Although this probability may seem already small, it would be a mistake to disregard even weaker interactions as irrelevant: including stretches with hybridization energy as weak as -8 kcal/mol (binding probability ≳ 1%) increases the total number of potentially hybridizing stretches up to ~500,000, and including interactions as weak as -7 kcal/mol (binding probability ≳ 0.02 %) increases the number to ~1,300,000. Even if binding events between weaker and weaker stretches are unlikely, their abundance grows so dramatically that these small binding probabilities end up becoming relevant. This is immediately apparent when comparing the effect of these different energy thresholds on the simulations’ outcome as shown in Fig. S1, where pushing the boundary to weaker interactions increases both the systems connectivity and pairings.

Even though we used a -7 kcal/mol cutoff to get rid of extremely weak interactions, this analysis suggests that including them could further increase the connectivity of the system, driving it towards more interactions and cluster formation. In this sense, our simulation represents a conservative estimate of the degree of intermolecular hybridization in the system.

1. **ViennaRNA accuracy**

In this work we have relied on ViennaRNA(2) to predict the folded structure of *E. coli* mRNAs. To determine whether these predictions could be used as proxies for *in vivo* mRNA accessibility, we compared computed values with experimental DMS-Seq data (3) retrieved from the RASP atlas(4) for a set of randomly picked *E. coli* sequences. Each entry allows to infer RNA accessibility by providing reactivities of cytosines and adenines to the alkylating agent DMS at the single nucleotide resolution.

DMS experiments generate a list of reactivities to the alkylating agent that can be normalized over a continuous interval from zero to one but unfortunately do not provide a binary output for paired/unpaired nucleotides. For any DMS experiment, a reactivity threshold has to be picked to determine what nucleotides can be considered DMS-paired (reactivity below the threshold) or DMS-unpaired (reactivity above the threshold).

To evaluate whether ViennaRNA agreed with experimental evaluation of mRNA structures *in vivo*, we computed the expected base-paired probability of our transcripts of choice and then split their nucleotides into “weakly paired” and “strongly paired” based on their *P_i_* values as previously defined.

For each subset of “weakly paired” and “strongly paired” nucleotides as predicted with ViennaRNA, we computed the fraction of bases whose state agrees with DMS assessment as we varied the DMS-reactivity threshold. This approach yields “True Paired Rate” vs “True Unpaired Rate” monotonously decreasing functions, highlighting the compromise between high sensitivity and specificity. The integral of the area for these curves (AUC) directly informs on the agreement between the two methods. In all cases analyzed here, we found that the AUC was larger than 0.5, with many cases showing exceptionally high concordance (Fig. S2).


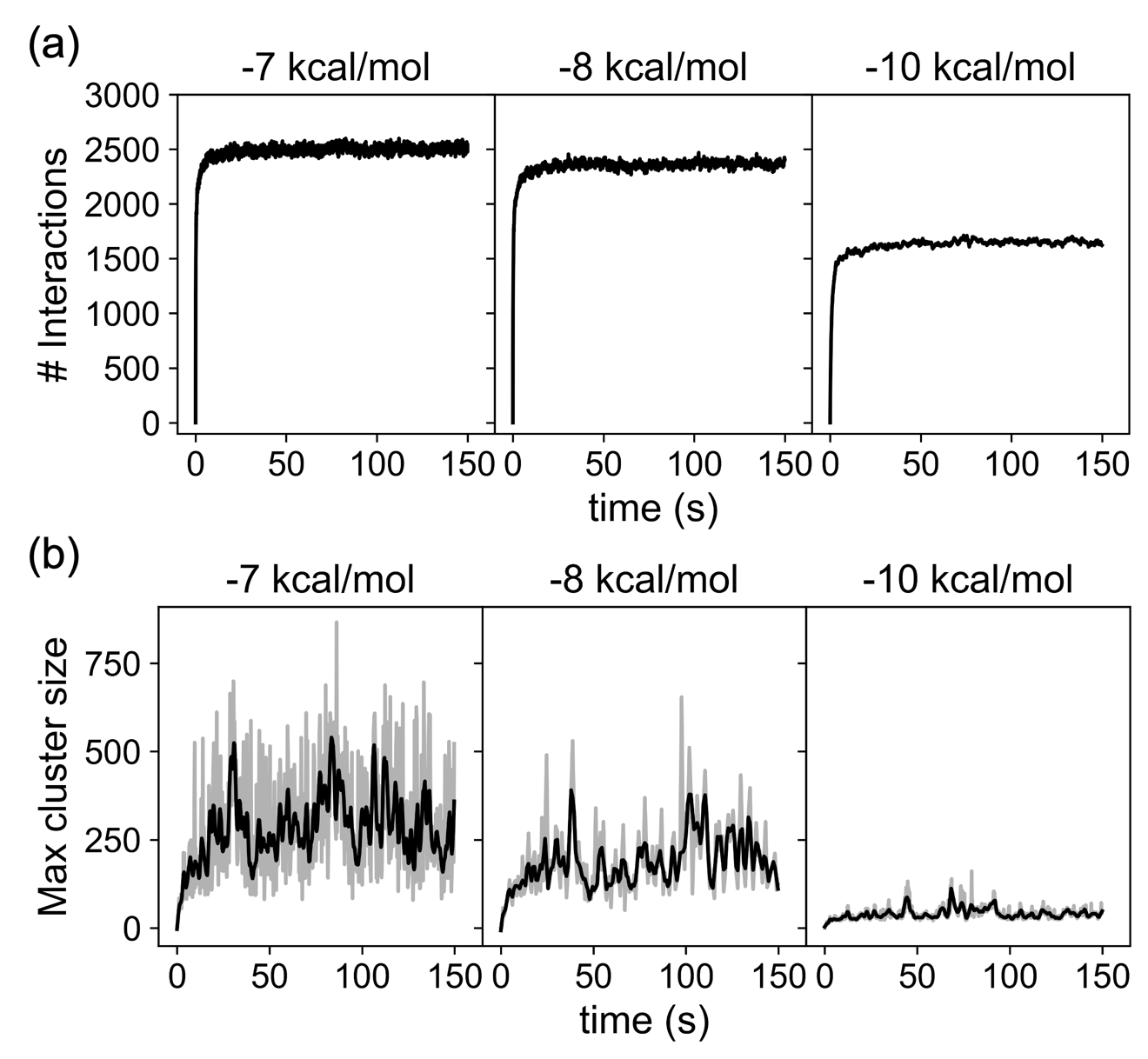


Fig. S1. Effect of different energy thresholds on the simulations’ outcome. The (a) total number of interactions and (b) largest cluster size decrease if only the most stable interactions are allowed in the simulation.


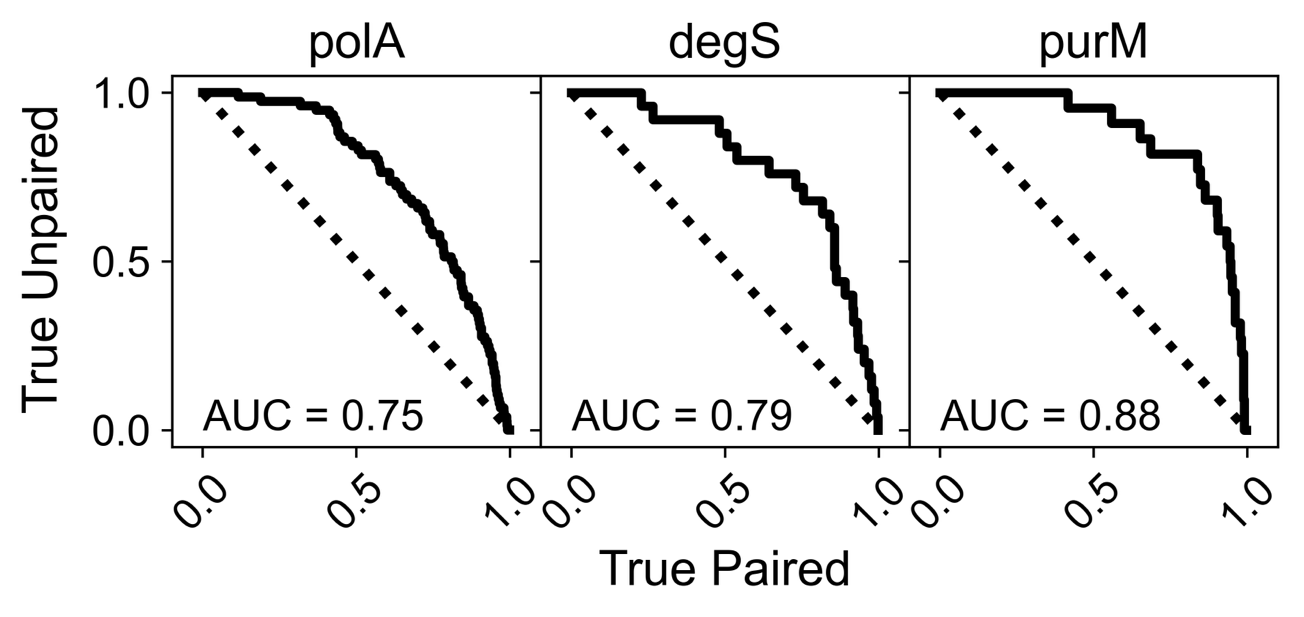


Fig. S2. Folding of *E. coli* mRNAs by ViennaRNA is in good agreement with experimental assessment of RNA accessibility by DMS-Seq. (a, b, c) Examples of sequences yielding high agreement between the two methods.

**SI References**

1. R. B. Martin, Comparisons of Indefinite Self-Association Models. *Chem. Rev.* **96**, 3043–3064 (1996).

2. R. Lorenz, *et al.*, ViennaRNA Package 2.0. *Algorithms Mol Biol* **6**, 26 (2011).

3. D. H. Burkhardt, *et al.*, Operon mRNAs are organized into ORF-centric structures that predict translation efficiency. *eLife* **6**, e22037 (2017).

4. P. Li, X. Zhou, K. Xu, Q. C. Zhang, RASP: an atlas of transcriptome-wide RNA secondary structure probing data. *Nucleic Acids Research* **49**, D183–D191 (2021).
